# The complete mitochondrial genome of the Emei black chicken (*Gallus gallus*) and its phylogenetic analyses

**DOI:** 10.1080/23802359.2020.1791019

**Published:** 2020-07-15

**Authors:** Jingjing Gu, Sheng Li

**Affiliations:** a College of Animal Science and Technology, Hunan Agricultural University, Changsha, China; b Hunan Key Laboratory for Genetic Improvement of Animals, Changsha, China; c Hunan Engineering Research Center of Poultry Production Safety, Changsha, China; d Maxun Biotechnology Institute, Changsha, China

**Keywords:** Mitochondrial genome, Emei black chicken, next generation sequencing

## Abstract

In this study, the complete mitochondrial genome of Emei black chicken (*Gallus gallus*) was obtained by using next generation sequencing method. The complete mitogenome sequence is 16,784 bp in length, containing 13 protein-coding genes, two ribosomal RNAs, 22 transfer RNA genes, and one control region. This work provides a valuable genetic resource of data for the *Gallus gallus* evolution study and contributes to the breeding improvement program of native chicken breeds.

Emei black chicken is an excellent dual-purpose type of indigenous chicken in the mountainous area around Sichuan Basin, China. The body size of Emei black chicken is large and the feathers are all black. Emei black chicken has strong disease resistance, good meat production and delicious meat quality. The long-term acclimation made the chicken resistant to crude feed, adaptive to cold growing environment, and suitable for high mountain free ranging. To understand its matrilineal genetic background and improve the breeding strategy, we collected the purebred Emei black chicken sample and sequenced its complete mitochondrial genome using high throughput sequencing technology. Emei black chicken sample was obtained in Emeishan City (29.60 N and 103.48 E), Sichuan Province, China. The total genomic DNA was extracted from the muscle specimen (Voucher No. EM150626) which stored at −80 °C in the Museum of Hunan provincial key laboratory for genetic improvement of domestic animal, Changsha, China. The genomic DNA was used as input material to generate sequencing libraries. The prepared libraries were then sequenced on Illumina Hiseq 2500 sequencer (San Diego, CA). In total, we generated 12.86 Gb raw data which been deposited in the NCBI Sequence Read Archive (SRA) with accession number SRX2188546. Using the sequence data we assembled the complete mitochondrial genome and annotated by tRNAscan-SE 2.0 (Chan and Lowe 2019) and MITOS (Bernt et al. 2013). The mitogenome sequence has been uploaded in the GenBank database with accession number MT555047.

The total length of the complete mitochondrial genome is 16,784 bp, with the base composition of 30.2% for adenine (A), 23.7% for thiamine (T), 32.5% for cytosine (C) and 13.5% for guanine (G). The complete mitogenome of this chicken breed exhibits a typical genetic structure of vertebrate organelle genome. It contains one noncoding control region (D-loop region), 22 transfer RNA genes (tRNAs), two ribosomal RNA genes (rRNAs), and 13 protein-coding genes (PCGs). Most genes including 12 PCGs, two rRNAs, and 14 tRNAs are encoded on the heavy chain. While only one PCG (*ND6*) and rest eight tRNAs are encoded on the light chain. Twelve of 13 PCGs initiated with an ATG start codon except for *COX1*, which began with GTG. Nine of the 13 PCGs use TAA as the stop codon. The *ND2* is stopped with TAG, the *COX1* is stopped with AGG and *COX3* together with *NAD4* are ended with incomplete stop codon T, which is the 5′ terminal of adjacent gene (Anderson et al. 1981). The longest protein-coding gene is *ND5*, which is 1833 bp; and the shortest one is the *ATP8*, which is 165 bp. The 12S- and 16S ribosomal RNA genes are 977 and 1552 bp long, respectively. The tRNA genes range from 66 to 76 bp in length. The non-coding control region of the Emei black chicken mitogenome is 1231 bp long, accounting for 7.33% of the total mitogenome length.

To investigate the phylogenetic position of Emei black chicken, we constructed the neighbor-joining (NJ) phylogenetic tree using Mega 7.0 (Kumar et al. 2016) with 1000 bootstrap replicates. Thirty-six complete mitogenomes of diverse chicken breeds and a Turkey (*Meleagris gallopavo*) as an outgroup were used to build the NJ tree. The result (Figure 1) shown that Emei black chicken and some chicken breeds grouped to one clade which indicated that Emei black chicken has the closest relationship with Hunan autochthonic, Huaiyang, Henan autochthonic, Nandan and Lverwu. This work provides a valuable genetic resource of data for the *Gallus gallus* evolution study and contributes to the breeding improvement program of native chicken breeds.

**Figure 1.**
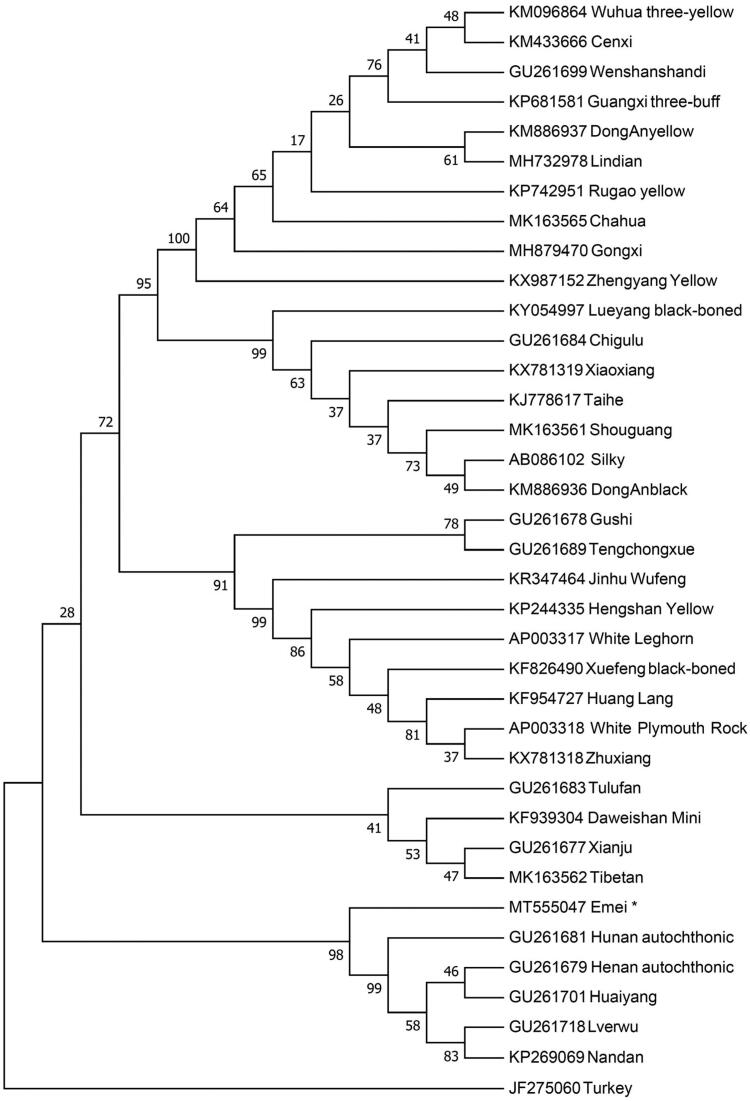
Neighbour-joining tree based on the complete mitochondrial DNA sequence of 36 chicken breeds and a Turkey as an outgroup. GenBank accession numbers are given before the species name.

## Data Availability

The sequence data that support the findings of this study are openly available in the NCBI Sequence Read Archive (SRA) at http://www.ncbi.nlm.nih.gov/sra/ with accession number SRX2188546. The complete mitochondrial genome of Emei black chicken (*Gallus gallus*) is openly available in GenBank at http://www.ncbi.nlm.nih.gov/genbank with accession number MT555047.
